# Secular trends and ethnic disparities in age at menarche among ethnic minority girls in China: a 35-year nationwide repeated cross-sectional study from 1985 to 2019

**DOI:** 10.7189/jogh.16.04221

**Published:** 2026-07-17

**Authors:** Di Shi, Yunfei Liu, Jiajia Dang, Shan Cai, Yaqi Wang, Jianhui Guo, Xinyao Lian, Shuyue Li, Junyu Lu, Tianyu Huang, Jiaxin Li, Ruolan Yang, Qiuyuan Chen, Peijin Hu, Jun Ma, Jing Li, Yi Song

**Affiliations:** 1 Institute of Child and Adolescent Health, School of Public Health, Peking University, Beijing, China; 2 National Health Commission Key Laboratory of Reproductive Health, Beijing, China

**Keywords:** age at menarche, ethnic minority, developmental trajectory, health equity, socioeconomic factors

## Abstract

**Background:**

Age at menarche (AAM) is a critical marker of female development and long-term health. While its global decline has been documented, its developmental trajectories and heterogeneity among China’s large ethnic minority populations remain poorly characterised. We sought to explore the temporal trends and differences in AAM between 26 major ethnic minority groups and their Han peers in China, and to examine the role of economic development in shaping these patterns.

**Methods:**

We retrieved data on 190,064 girls from seven waves (1985–2019) of the Chinese National Survey on Students’ Constitution and Health, covering 26 minority groups and local Han peers across 13 provincial-level regions. We estimated the median AAM and its 95% confidence interval (CI) *via* probit regression based on *status quo* menarcheal data. We assessed intergroup differences using Z-tests, identified distinct trajectories of differences in AAM between 25 minority groups and their local Han peers through K-means clustering; and examined associations between AAM and log-transformed prefecture-level *per capita* GDP using quadratic regression models.

**Results:**

The median AAM among minority girls in 2019 was 12.54 years, later than that of Han girls (12.05 years), with an overall difference of 0.49 years and substantial interethnic heterogeneity. The AAM declined across all minority groups from 1985 to 2019, albeit at different rates. Three trajectories of minority–Han differences were identified: a ‘rapid catch-up’ pattern (n/N = 13/25 groups), an ‘emerging divergence’ pattern (n/N = 8/25 groups), and a ‘fluctuating/stable’ pattern (n/N = 4/25 groups). These clusters exhibited a clear gradient in regional economic development. Economic growth showed a nonlinear association with AAM, with stronger declines at lower development levels and a plateau at higher levels.

**Conclusions:**

Although regional economic development was associated with AAM among ethnic minorities in China, convergence with Han peers remains uneven. The nonlinear association with economic growth suggests diminishing returns of development, underscoring the need for targeted public health strategies addressing both persistent deprivation and modernisation-related risks.

Systematic investigations of biological development trajectories among ethnic minority adolescent populations are crucial for advancing health equity within the context of the Sustainable Development Goals [1,2]. Age at menarche (AAM) is a widely used marker of female pubertal development and is associated with socioeconomic, nutritional, environmental, and broader ecological conditions [3,4]. Long-term declines in AAM have been reported in many populations since the intensification of industrialisation in the 19th century, with decreases of approximately 2–3 months per decade reported in several high-income settings [5,6]. China has experienced even steeper declines over the past 50 years, largely attributed to improved nutrition, rising obesity prevalence, and heightened exposure to endocrine-disrupting chemicals [7–10]. However, research has predominantly focused on single-ethnic or homogeneous populations, overlooking ethnic variations in developmental trajectories. Because ethnic groups differ in their geographic distribution, socioeconomic conditions, dietary patterns, and cultural contexts, their long-term trajectories of pubertal development may also differ.

China officially recognises 56 ethnic groups, comprising the Han majority and 55 ethnic minority groups distributed across diverse ecological and economic regions [11]. Despite this diversity, minority health remains underrepresented in developmental research. Previous studies indicate that the median AAM among Han Chinese girls declined from 13.3 years in 1985 to 12.05 years in 2019, at a rate of 4.3 months per decade – a trend widely attributed to rapid socioeconomic growth [7]. 

However, it remains unclear whether comparable long-term declines occurred consistently across all major ethnic minority groups, how differences relative to local Han populations changed over time, and to what extent regional economic conditions were associated with these patterns. Data up to 2014 suggest narrowing AAM gaps between Han and 17 minority groups, although the magnitude and direction of change varied across groups and survey years [12]. Existing evidence largely extends only to 2014, leaving more recent trends through 2019 insufficiently characterised [12].

Leveraging data from seven waves (1985–2019) of the Chinese National Survey on Students’ Constitution and Health (CNSSCH) [13], we aimed to describe AAM among girls from 26 ethnic minority groups in 2019 and quantify differences relative to their local Han peers; examine the long-term trends in AAM and changes (convergence or divergence) in differences between minority and Han ethnic groups from 1985 to 2019; and assess the associations of both AAM levels and trajectories of minority–Han differences with regional economic development. We hypothesised that AAM would decline across most ethnic minority groups, while the magnitude and direction of minority–Han differences would vary across groups and over time. We further hypothesised that economic development would be nonlinearly associated with AAM and with changes in differences between minority and Han populations.

## METHODS

As this was a secondary analysis of data from a repeated cross-sectional study, we report our study according to the STROBE and GRABDROP guidelines (Checklists S1 and S2 in the **Online Supplementary Document**).

### Study sample

We retrieved data on 190,064 girls from the 1985, 1990, 2000, 2005, 2010, 2014, and 2019 CNSSCH, the largest repeated cross-sectional survey of child and adolescent health in China. Conducted every five years across 31 provincial-level regions in mainland China (excluding Taiwan, Hong Kong, and Macau), the CNSSCH targets individuals aged 6–22 years, with the majority being of Han ethnicity. The sampling protocol, detailed in prior publications [10,14], initially employed stratified cluster sampling to select regions of high, medium, and low economic development within each province. Subsequent survey waves generally retained the same survey schools, indicators, and standardised procedures to enhance comparability over time. Participants were required to have resided locally for at least one year, while individuals with severe physical or mental illnesses were excluded (Figure S1 in the **Online Supplementary Document**).

The CNSSCH included 26 ethnic minorities from 13 provincial-level regions with significant minority populations: Mongolian (Inner Mongolia), Hui (Ningxia), Tibetan (Tibet), Uighur, Kazakh/Hasake and Kirghiz (Xinjiang); Miao, Buyi, Dong, and Shui (Guizhou); Yi and Qiang (Sichuan); Zhuang and Yao (Guangxi); Bai, Hani, Dai, Lisu, Wa/Va, and Naxi/Nakhi (Yunnan); Korean (Jilin); Dongxiang (Gansu); Tu and Sala (Qinghai); Tujia (Hunan); and Li (Hainan). In Tibet, only Tibetan students were surveyed, while other provinces included both minority and local Han populations. Data were unavailable for several ethnic group–survey wave combinations because the corresponding groups were not included in those survey waves: Yi (1995, 2000, 2005), Kazakh (2000, 2005), Shui (1985–2000), Dongxiang (1985–2005), Kirghiz (2005, 2014), Mongolian (1995), Uighur (2005), Naxi (2000), Dai (1995), Li (2005), Hani (2000), Lisu (2000), Wa (2000), Bai (1995), and Tujia (1995).

### Measures

The analytic sample comprised 190,064 ethnic minority girls aged 9–18 years across the seven survey waves (Table S1 in the **Online Supplementary Document**). 

For the CNSSCH, trained female physicians conducted individual face-to-face interviews to assess whether each participant had experienced menarche. When necessary, they provided a brief standardised explanation of menarche before recording the response. Responses were recorded as a binary variable (yes/no), with participants who declined to answer being excluded from our analysis.

We obtained prefecture-level *per capita* gross domestic product (GDP) data corresponding to the survey locations from the statistical yearbooks and statistical bulletins (Table S2 in the **Online Supplementary Document**). For each ethnic group and survey year, we identified the prefecture-level cities containing the fixed survey counties or districts and calculated the unweighted arithmetic mean of the available *per capita* GDP values across the unique cities. We expressed GDP in RMB and log-transformed it before analysis. Ethnic group–year observations with unavailable GDP data were excluded from the corresponding regression analyses, and no imputation was performed. Because prefecture-level GDP data were not available for 1985, the economic analyses were restricted to the 1995–2019 survey waves.

### Statistical analysis

Because the CNSSCH recorded current menarcheal status at the time of examination rather than the exact age at menarche, individual AAM values were not directly observed. We therefore estimated median AAM and its 95% confidence interval (CI) using probit regression based on the *status quo* method. We stratified participants into one-year age intervals (*e.g.* 9.0–9.9, 10.0–10.9 years). We transformed the cumulative proportion of girls attaining menarche within each age stratum *via* probit regression and derived the median AAM (with 95% CI) as the age at which 50% of the population was estimated to have reached menarche, based on the fitted normal distribution. For the overall 2019 estimates, all eligible ethnic minority girls were pooled and analysed in a single probit model, while eligible Han girls were pooled and analysed separately. The validity of this method for estimating AAM has been supported by prior pubertal timing studies using the same probit framework [10,12,15,16].

We employed Z-tests to compare AAM differences between ethnic minority groups and their Han Chinese counterparts within the same provinces. To account for multiple comparisons across the 25 ethnic minority–Han comparisons conducted in 2019, the resulting two-sided P values were adjusted using the Benjamini–Hochberg false discovery rate procedure. A false discovery rate-adjusted *P*-value (*q*-value) of <0.05 was considered statistically significant. 

We used quadratic regression models to examine the nonlinear association between log-transformed *per capita* GDP and median AAM within each survey year, with model fit assessed by *R*^2^ and the F-statistic *P*-value. We specified the within-year model as:

*AAM *= *β*0 + *β*1*log*(*GDP*) + *β*2(*log*(*GDP*))^2^ + *ε*

Additionally, to estimate the overall association while adjusting for temporal trends and regional differences, we fitted a multivariable OLS regression model using pooled data (1995–2019):

*AAM* = *β*0 + *β*1*log*(*GDP*) + *β*2(*log*(*GDP*))^2^ + *Σγt*(*survey year*)*t* + *Σλp*(*province*)*p* + *ε*

Here, *survey year* and *province* were modeled as fixed effects. Due to incomplete economic data, the 1985 survey was excluded from these analyses.

To systematically identify long-term patterns in ethnic disparities, we employed a K-means clustering algorithm. For each ethnic minority group, we extracted four key features describing the evolution of the AAM gap relative to Han peers: the initial gap (*δ*_initial), defined as the first observed minority–Han AAM gap for that ethnic group; the final gap (*δ*_final), defined as the last observed minority–Han AAM gap; the total change in the gap (*δ*_change = *δ*_final − *δ*_initial); and the recent slope of the gap (calculated *via* linear regression from 2010 to 2019 using available observations). These features were standardised using Z-score normalisation to ensure comparability. We performed K-means clustering and subsequently merged the clusters into three distinct interpretative trajectories based on their feature characteristics: ‘rapid catch-up’ (significant negative *δ*_change, indicating gap closure), ‘emerging divergence’ (positive *δ*_change or positive recent slope, indicating widening gaps), and ‘fluctuating/stable’ (minimal overall change, with fluctuating trends). Because only Tibetan students were surveyed in Tibet in the CNSSCHs, with no corresponding local Han sample, the Tibetan group was included in descriptive analyses but excluded from minority–Han disparity clustering; therefore, clustering was conducted for 25 ethnic groups. Sampling weights and school-level clustering were not incorporated into the analyses.

We compared differences in 2019 *per capita* GDP across the three convergence categories using the non-parametric Kruskal–Wallis test, followed by *post-hoc* pairwise comparisons with Dunn’s test. A two-sided *P*-value <0.05 was considered statistically significant. All analyses were performed using Python, version 3.12 (Python Software Foundation, Wilmington, Delaware, USA), and *R*, version 4.3.2 (R Foundation for Statistical Computing, Vienna, Austria).

## RESULTS

### Menarche status in ethnic minorities in 2019

**Figure 1:**
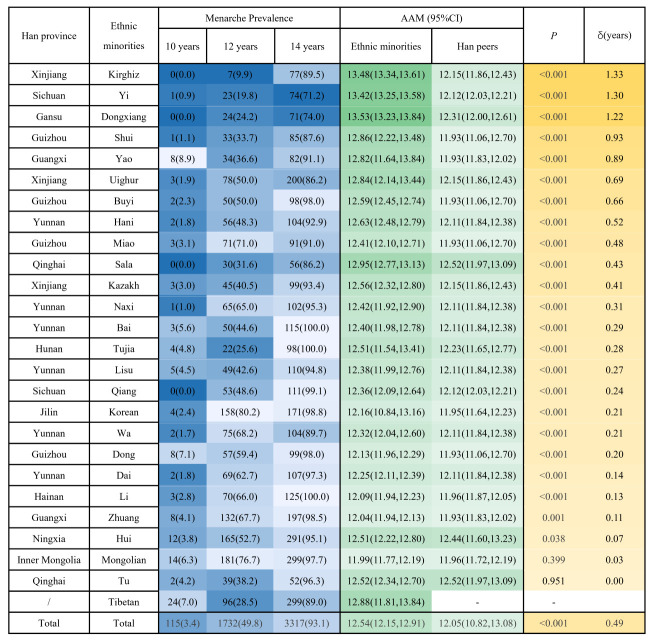
Age-specific menarche incidence rates among ethnic minorities and comparison with the Han peers regarding AAM in 2019. Columns represent the prevalence of menarche at ages 10, 12, and 14 years, with darker blue shading indicating lower prevalence rates. The median AAM (95% CI) for each ethnic minority group and their local Han peers was estimated using probit regression. The disparity (*δ*) denotes the difference in years between the minority and Han AAM (positive values indicate minority delay). *P*-values were derived from Z-tests comparing the two groups. AAM – age at menarche, CI – confidence interval.

The median AAM across 25 large-population ethnic minority groups in 2019 was 12.54 years (95% CI = 12.15–12.91), significantly later than that of their Han peers residing in the same provinces (12.05 years, 95% CI = 10.82–13.08), with an overall difference (*δ*) of 0.49 years (*P* < 0.001) (**Figure 1**). Age-specific incidence rates indicated that by age 10, only 3.4% of minority girls had attained menarche. The highest incidence rates at age 10 were observed among Yao (8.9%, Guangxi), Tibetan (7.0%, Tibet), and Mongolian (6.3%, Inner Mongolia) girls. The cumulative incidence by the age of 12 years rose to 49.8%, with the highest rates found in Korean (80.2%, Jilin), Mongolian (76.7%, Inner Mongolia), and Miao (71.0%, Guizhou) ethnicities and the lowest in Kirghiz (9.9%, Xinjiang), Yi (19.8%, Sichuan) and Dongxiang (24.2%, Gansu) groups. By the age of 14 years, 93.1% of all minority girls had reached menarche.

We observed substantial heterogeneity in AAM differences relative to local Han populations. Notably, the Kirghiz (*δ* = 1.33 years, *P* < 0.001, Xinjiang), Yi (*δ* = 1.30 years, *P* < 0.001, Sichuan), and Dongxiang (*δ* = 1.22 years, *P* < 0.001, Gansu) minorities exhibited the most pronounced delays. However, the AAM gap with local Han peers had effectively closed for specific groups. We found no statistically significant difference between the Mongolian (*δ* = 0.03 years, *P* = 0.399, Inner Mongolia) and Tu (*δ* = 0.00 years, *P* = 0.951, Qinghai) girls and their Han counterparts. After applying the Benjamini–Hochberg false discovery rate correction, 23 of the 25 minority–Han comparisons remained statistically significant (*q* < 0.05), meaning all except for the differences for Mongolian girls (*q* = 0.416) and Tu girls (*q* = 0.951).

### Long-term trends in age at menarche and changes in minority–Han difference

We observed an overall long-term decline across all 26 ethnic minority groups, although the trajectories were not monotonic in every group (Figures S2 and S3 in the **Online Supplementary Document**). The most rapid decreases occurred among the Dongxiang (11.1 months per decade, Gansu), Lisu (8.6 months per decade, Yunnan), and Dong (7.5 months per decade, Guizhou) minorities.

**Figure 2:**
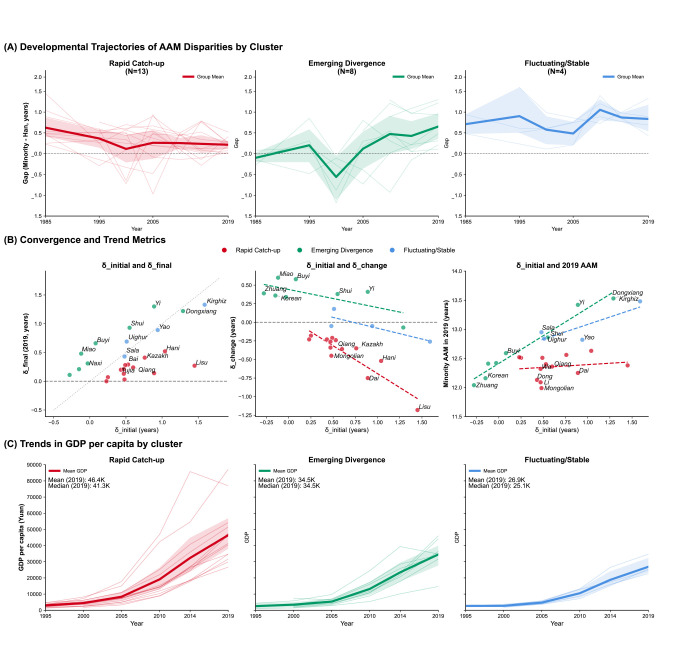
Characterisation of developmental trajectories in minority-Han AAM disparities by clustering. **Panel A.** Three identified developmental trajectories of the AAM gap relative to Han peers for 25 ethnic groups: ‘rapid catch-up’ (red), ‘emerging divergence’ (green), and ‘fluctuating/stable’ (blue). Lines represent the smoothed trends for each cluster. **Panel B.** Validation of clustering patterns showing the relationship between the initial gap (*δ*_initial), the final gap (*δ*_final), the total change (*δ*_change = *δ*_final − *δ*_initial) and the median AAM in 2019. The scatter plots highlight the distinct separation of the three clusters based on disparity evolution features. **Panel C.** The prefecture-level log-transformed *per capita* GDP across the three clusters from 1995 to 2019. The Tibetan group was excluded from this analysis due to the absence of local Han reference data.

Using K-means clustering based on the magnitude and rate of change in minority–Han differences, we identified three distinct developmental trajectories among the 25 ethnic groups with sufficient data (**Figure 2**, Panel A; Table S3 in the **Online Supplementary Document**). The dominant pattern was ‘rapid catch-up’ (n = 13, 52.0%), characterised by a substantial reduction in the AAM gap relative to Han peers over time. This cluster included groups such as the Lisu, Hani, and Mongolian minorities, where the initial large gaps have significantly narrowed or closed. The second pattern was ‘emerging divergence’ (n = 8, 32.0%), which included the Miao, Buyi, Zhuang, and other ethnic subgroups. These ethnicities exhibited a trend of widening disparities, particularly in the recent decade (2010–2019), despite some earlier declines. The third pattern was ‘fluctuating/stable’ (n = 4, 16.0%), comprising the Yao, Kirghiz, Sala, and Uighur ethnicities. These minorities maintained persistent gaps or showed fluctuating trends without a clear direction of convergence.

The cluster-specific patterns were further illustrated by the trajectory metrics (**Figure 2**, Panel B). First, plotting *δ*_initial against *δ*_final showed visual separation among the three clusters. Second, groups with larger initial differences tended to show greater reductions in the gap over time. Third, the relationship between *δ*_initial and 2019 AAM differed across clusters; the ‘rapid catch-up’ group generally showed lower AAM in 2019 despite larger initial differences in some ethnic groups.

### Associations of regional *per capita* GDP with median age at menarche and minority–Han difference trajectories

**Figure 3:**
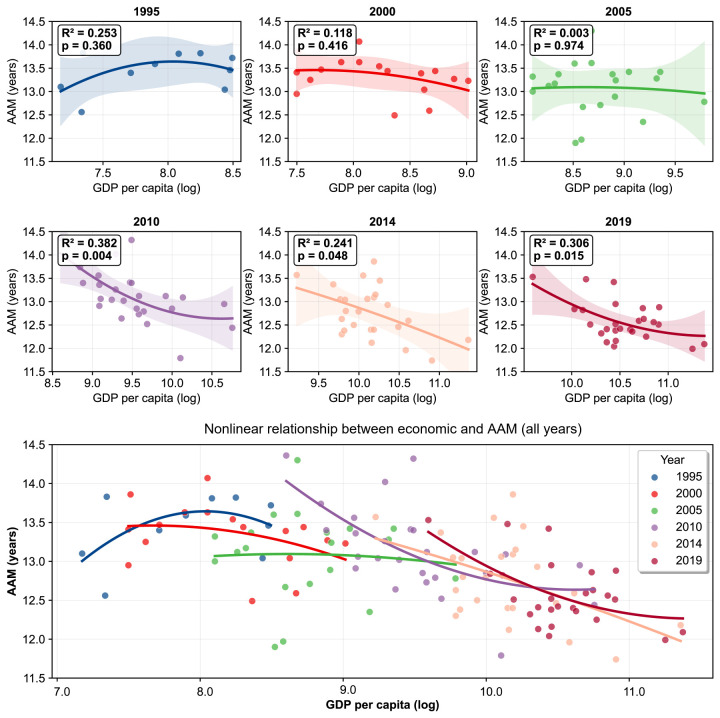
Associations between median age at menarche and log-transformed regional *per capita* GDP, 1995–2019. Scatter plots display the relationship between district-level log-transformed per capita GDP (x-axis) and the median AAM of ethnic minority groups (y-axis) across different survey years (1995–2019). The solid curves represent the fitted quadratic regression models, with shaded areas indicating 95% CIs. *R*^2^ values describe model fit, and the displayed *P*-values represent the overall F-tests for the corresponding year-specific models.

In the pooled model adjusted for survey year and province, the quadratic term for log-transformed *per capita* GDP was statistically significant. The pooled model had an adjusted *R*^2^ of 0.566 and was statistically significant overall (*P* < 0.001), with a significant negative quadratic term (coefficient = −0.092; *P* = 0.032) (**Figure 3**; Table S4 in the **Online Supplementary Document**).

However, the shape of this association exhibited a distinct temporal shift. In the earliest survey (1995), the relationship followed an inverted U-shaped pattern with a negative quadratic coefficient of −0.858, though the association was not statistically significant (*P* = 0.360). In contrast, the association became stronger and statistically significant in the recent surveys from 2010 (*R*^2^ = 0.382; *P* = 0.004) and 2019 (*R*^2^ = 0.306; *P* = 0.015), with the quadratic coefficients in these later years shifting to positive values of 0.341 and 0.329, respectively. The fitted curves suggested possible attenuation at higher GDP levels; however, this pattern should be interpreted descriptively because the quadratic terms in the individual 2010 and 2019 models were not statistically significant (**Figure 3**).

This economic gradient was systematically reflected in the difference trajectories identified (**Figure 2**, Panel C; Table S5 in the **Online Supplementary Document**). The ‘rapid catch-up’ cluster was situated in regions with the highest economic development (2019 mean GDP = CNY 46 417), significantly higher than the ‘emerging divergence’ cluster (2019 mean GDP = CNY 34 509) and the ‘fluctuating/stable’ cluster, which resided in the least developed regions (2019 mean GDP = CNY 26 903). Overall, a clear economic gradient was evident across the identified trajectories: the ‘rapid catch-up’ cluster coincided with the highest regional economic levels, whereas the ‘emerging divergence’ and ‘fluctuating/stable’ clusters corresponded to regions with lower *per capita* GDP.

## DISCUSSION

Using seven waves of a nationwide repeated cross-sectional survey, we examined long-term AAM trajectories in an analytic sample of 190,064 girls from 26 ethnic minority groups in China; 25 groups with local Han comparators were included in the clustering analysis. Overall, we observed a significant long-term decline in AAM and a narrowing difference between minority and Han girls, mirroring global trends in low- and middle-income countries [17]. However, this aggregate convergence masks significant ethnic heterogeneity. Through multi-dimensional clustering, we identified three distinct trajectories: ‘rapid catch-up’, ‘emerging divergence’, and ‘fluctuating/stable’. While most groups (*e.g.* Lisu, Hani) achieved a rapid closure of the developmental gap, specific populations (*e.g.* Yi, Kirghiz) exhibited persistent or widening differences. This divergence not only reflects the uneven regional development during China’s economic transition, but also suggests heterogeneity in the contextual factors associated with adolescent development [18].

The long-term decline in AAM carries a dual significance for public health. For the ‘rapid catch-up’ groups, earlier menarcheal timing may be consistent with improvements in living conditions, food security, and nutritional status over recent decades [19,20]. However, these pathways were not measured directly in the present study and should be considered possible explanations rather than established mechanisms. Conversely, for groups where AAM has reached historically low levels (*e.g.* Korean minorities), the continued trend toward earlier maturation may no longer signal optimal nutrition alone, but may also be related to the accumulation of modern lifestyle risks [21]. Previous studies have reported associations of childhood adiposity, obesogenic environments, and exposure to endocrine-disrupting chemicals with earlier pubertal timing [9,10,22]. Thus, the long-term trend may represent a transition from the burden of ‘undernutrition’ to risks of ‘overnutrition and environmental exposure’, underscoring the need to balance developmental catch-up with the prevention of long-term risks associated with early menarche, such as cardiovascular disease and breast cancer [23,24].

Despite the overall positive trends, the persistence of ‘fluctuating/stable’ and ‘emerging divergence’ clusters highlights entrenched health inequalities. The developmental lag remains pronounced among the Yi (Sichuan) and Kirghiz (Xinjiang) populations, with gaps exceeding 1.2 years relative to Han peers. This pattern may be related to the intersection of geographic isolation and structural barriers [25]. Although regional GDP has grown, the accessibility of public health resources and the ‘last mile’ delivery of quality nutrition may remain constrained by geography [26,27]. Furthermore, the widespread phenomenon of ‘left-behind children’ due to labour migration may exacerbate these disparities. The lack of parental care may be associated not only with dietary monotony (micronutrient deficiencies), but also with chronic psychosocial stress, which has been linked to altered neuroendocrine regulation of the hypothalamic-pituitary-gonadal axis, leading to delayed maturation in resource-poor settings [28–30]. These persistent disparities may represent the biological embedding of structural inequalities [31].

We further determined a significant nonlinear association between regional economic development and AAM. In the pooled model, regional per capita GDP was nonlinearly associated with AAM after adjustment for survey year and province. Previous studies have similarly reported that associations between economic development and population health may vary across development levels [32,33]. In the early stages of development (1995–2005), GDP growth appeared to be an important correlate of alleviating subsistence constraints and promoting catch-up. However, the year-specific fitted curves suggested that the association may attenuate at higher GDP levels. Because the quadratic terms were not statistically significant in the individual 2010 and 2019 models, the apparent plateau should be interpreted cautiously. Similar nonlinear or threshold-like associations between economic development and population health have been reported in other contexts [34,35]. In these settings, the primary determinants of health may shift from material sufficiency to allocative efficiency, family environment, and health literacy [36,37]. Consequently, addressing remaining health disparities in developed regions may require targeted structural interventions rather than broad economic stimulation. These findings suggest that adolescent health strategies in ethnic minority regions may need to move beyond broad economic development alone and incorporate more targeted approaches, including improved access to health services, school-based puberty education, and monitoring of lifestyle-related developmental risks.

The strength of this study lies in its 35-year repeated cross-sectional design and large multi-ethnic sample, which allowed us to characterise long-term developmental patterns across diverse ethnic minority populations in China. The use of K-means clustering provided an empirical framework for identifying heterogeneous trajectories in minority–Han differences. However, several limitations should be noted. First, the repeated cross-sectional design precludes causal inference. Second, individual-level data on dietary intake, adiposity, environmental exposures, and psychosocial stress were unavailable, preventing us from examining specific pathways underlying the observed patterns. Finally, although the *status quo* method reduces reliance on recalling exact age at menarche, self-reported menarcheal status may still be affected by misunderstanding or reporting error.

## CONCLUSIONS

The median age at menarche among ethnic minority girls in China has declined substantially over the past 35 years, accompanied by an overall narrowing of differences relative to local Han peers. However, this convergence was uneven, with some ethnic groups showing persistent delays or emerging divergence. Regional economic development was nonlinearly associated with age at menarche, suggesting that the association between economic growth and developmental timing may attenuate at higher development levels. These findings highlight the need for equity-oriented adolescent health strategies that address persistent developmental disadvantages in less-developed regions while also monitoring potential risks associated with rapid socioeconomic and lifestyle transitions.

## Additional material


Online Supplementary Document
