# Supplementary material for: Secular trends and ethnic disparities in age at menarche among ethnic minority girls in China: a 35-year nationwide repeated cross-sectional study from 1985 to 2019
Source: J Glob Health. 2026 Jul 17;16:04221. doi: 10.7189/jogh.16.04221 (PMC13377758; doi:10.7189/jogh.16.04221)
Supplement: Online Supplementary Document [file jogh-16-04221-s001.pdf]

Supplement to: Shi D, Liu Y, Dang J, Cai S, Wang Y, Guo J, Lian X, Li S, Lu J, Huang T, Li J, Yang R, Chen Q, Hu P, Ma J, Li J, Song Y. Secular trends and ethnic disparities in age at menarche among ethnic minority girls in China: a 35-year nationwide repeated cross-sectional from 1985 to 2019. J Glob Health. 2026;16:04221.

Table S1 Sample sizes across survey years of 26 ethnic minorities

	**1985**	**1995**	**2000**	**2005**	**2010**	**2014**	**2019**
**Mongolian**	1,600	–	2,392	1,829	1551	2116	2543
**Hui**	1,494	2,398	1,999	2,045	2147	2334	2922
**Tibetan**	769	1,197	1,144	999	1047	1006	3318
**Uighur**	1,399	2,400	1,001	–	1893	1912	1833
**Miao**	696	1,101	1,720	999	992	997	995
**Yi**	865	–	–	–	1175	1195	1079
**Zhuang**	1,326	2,355	1,998	846	1943	2087	1890
**Buyi**	1,184	1,181	982	973	996	979	949
**Korean**	1,900	2,397	1,970	869	2151	1856	1560
**Dong**	723	1,200	931	998	983	998	984
**Yao**	616	1,199	1,001	989	859	957	846
**Bai**	800	–	1,001	1,100	1072	1091	967
**Tujia**	500	–	998	1,015	1016	1067	1018
**Hani**	734	1,154	–	1,074	1094	1099	1106
**Kazakh**	860	1,199	–	–	1200	1192	1041
**Dai**	609	–	972	1,085	1064	1097	1103
**Li**	700	1,200	909	–	865	1279	1123
**Lisu**	728	1,106	–	1,087	1027	1097	1139
**Wa**	613	1,090	–	1,098	1076	1097	1155
**Shui**	–	–	–	982	998	999	981
**Dongxiang**	–	–	–	–	937	1054	938
**Naxi**	700	1,200	–	1,096	1094	1040	1055
**Kirghiz**	449	1,200	500	–	1225	–	953
**Tu**	1,103	600	1,001	1,059	1085	1090	692
**Qiang**	861	1,232	848	1,170	1149	1151	1112
**Sala**	224	600	999	1,051	1072	1081	988
**Total**	21,453	26,009	22,366	22,364	31,711	31,871	34,290

Table S2 Statistical sources used for regional per capita GDP data

Ethnic group	Prefecture-level city/prefecture(s)	Statistical yearbook(s) or bulletin(s)
Mongolian	Xing'an League; Hohhot; Bayannur; Tongliao; Ordos; Xilingol League	China City Statistical Yearbook; Inner Mongolia Statistical Yearbook; Hohhot Statistical Yearbook; Tongliao Statistical Yearbook; Ordos Statistical Yearbook; Xilingol League Statistical Yearbook; Bayannur Statistical Communiqué; Inner Mongolia 1949–2009
Hui	Zhongwei; Wuzhong; Guyuan; Yinchuan	China City Statistical Yearbook; Ningxia Statistical Yearbook; Yinchuan Statistical Yearbook; Zhongwei Statistical Communiqué
Tibetan	Lhasa; Qamdo; Nyingchi; Nagqu	China City Statistical Yearbook; Lhasa Statistical Yearbook
Uighur	Kashgar Prefecture	Xinjiang Statistical Yearbook
Miao	Qiandongnan Miao and Dong Autonomous Prefecture	Qiandongnan Statistical Communiqué
Yi	Liangshan Yi Autonomous Prefecture	Sichuan Statistical Yearbook
Zhuang	Baise	China City Statistical Yearbook; Guangxi Statistical Yearbook
Buyi	Qianxinan Buyi and Miao Autonomous Prefecture	Qianxinan Statistical Communiqué
Korean	Yanbian Korean Autonomous Prefecture	Jilin Statistical Yearbook
Dong	Qiandongnan Miao and Dong Autonomous Prefecture	Qiandongnan Statistical Communiqué
Yao	Hechi	China City Statistical Yearbook; Hechi Statistical Yearbook
Bai	Dali Bai Autonomous Prefecture; Qujing	China City Statistical Yearbook; Yunnan Statistical Yearbook
Tujia	Xiangxi Tujia and Miao Autonomous Prefecture	China Regional Economic Statistical Yearbook; Hunan Statistical Yearbook; Xiangxi Statistical Communiqué
Hani	Honghe Hani and Yi Autonomous Prefecture	Yunnan Statistical Yearbook
Kazakh	Altay Prefecture	Xinjiang Statistical Yearbook; Altay Prefecture Statistical Yearbook
Dai	Xishuangbanna Dai Autonomous Prefecture	Yunnan Statistical Yearbook
Li	Sanya	China City Statistical Yearbook; Sanya Statistical Yearbook

Lisu	Nujiang Lisu Autonomous Prefecture	Yunnan Statistical Yearbook
Wa	Lincang	Lincang Statistical Communiqué
Shui	Qiannan Buyi and Miao Autonomous Prefecture	Qiannan Statistical Communiqué
Dongxiang	Linxia Hui Autonomous Prefecture	China Regional Economic Statistical Yearbook; Gansu Yearbook; Gansu Development Yearbook; Gansu 1949–2009
Naxi	Lijiang	China City Statistical Yearbook; Yunnan Statistical Yearbook
Kirghiz	Kizilsu Kirghiz Autonomous Prefecture	Xinjiang Statistical Yearbook
Tu	Haidong; Hainan Tibetan Autonomous Prefecture	China City Statistical Yearbook; China Regional Economic Statistical Yearbook; Qinghai Statistical Yearbook
Qiang	Aba Tibetan and Qiang Autonomous Prefecture	Sichuan Statistical Yearbook
Sala	Haidong; Hainan Tibetan Autonomous Prefecture	China City Statistical Yearbook; China Regional Economic Statistical Yearbook; Qinghai Statistical Yearbook

Figure S1. Geographical distribution of the study sample across provinces and participant flowchart

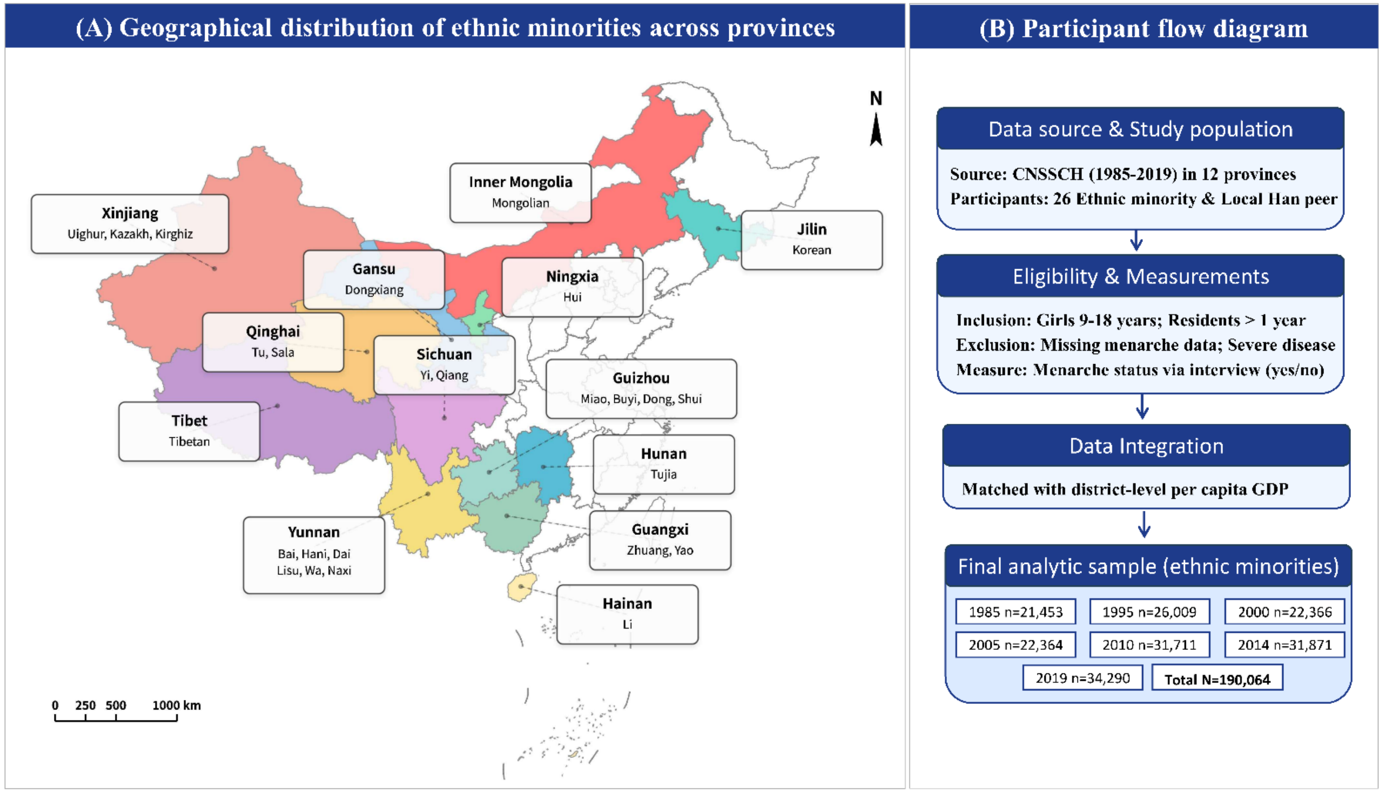

Figure S2. Twenty-six ethnic minorities and comparisons with local Han populations in China, 1985–2019.

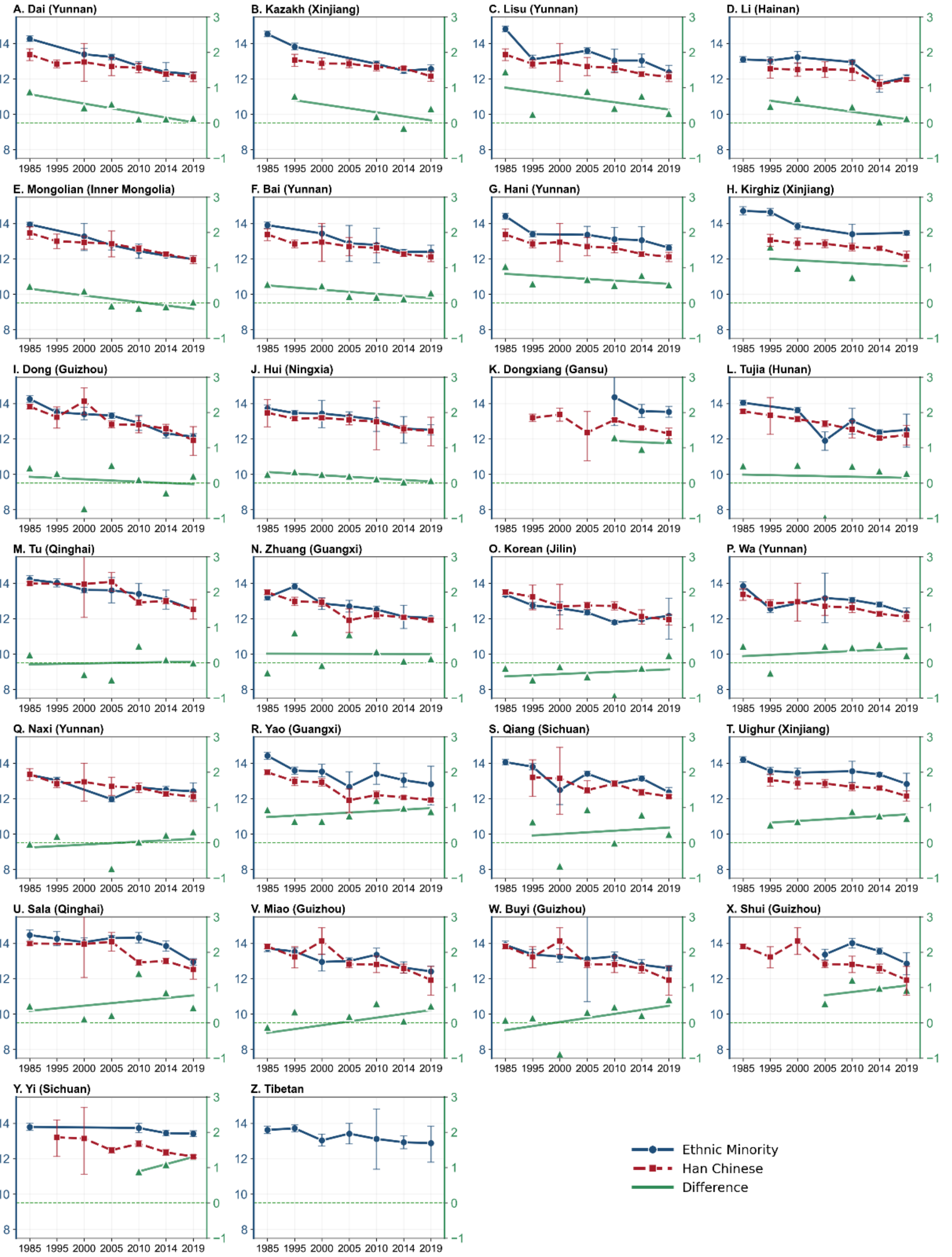

Note: Each panel represents one of the 26 ethnic minority groups. The blue left axis represents the median AAM of the ethnic minority girls, while the red dashed lines (left axis) represent the local Han peers. The green right axis depicts the difference (δ) trend, calculated as Minority AAM minus Han AAM. Error bars indicate 95% confidence intervals. Data for certain years were unavailable for specific groups (e.g. Yi, Shui).

Figure S3 Long-term trends in AAM among 26 ethnic minority groups

	AAM Decadal Decline Rate (months/decade)	1985	1995	2000	2005	2010	2014	2019
Dongxiang	-11.1	-	-	-	-	14.36(12.75,16.23)	13.57(13.22,13.95)	13.53(13.23,13.84)
Lisu	-8.6	14.83(14.66,14.99)	13.10(12.88,13.34)	-	13.60(13.43,13.76)	13.04(12.39,13.68)	13.04(12.67,13.40)	12.38(11.99,12.76)
Dong	-7.5	14.26(14.07,14.45)	13.50(13.31,13.68)	13.41(13.08,13.80)	13.32(13.16,13.48)	12.91(12.48,13.34)	12.30(12.05,12.55)	12.13(11.96,12.29)
Dai	-7.1	14.27(14.12,14.43)	-	13.39(13.09,13.72)	13.24(13.08,13.39)	12.73(12.47,12.98)	12.40(11.87,12.92)	12.25(12.11,12.39)
Kazakh	-7	14.54(14.39,14.69)	13.82(13.64,14.02)	-	-	12.85(12.70,13.00)	12.46(12.32,12.60)	12.56(12.32,12.80)
Mongolian	-6.9	13.95(13.82,14.07)	-	13.27(12.47,14.01)	12.78(12.51,13.04)	12.44(12.03,12.84)	12.18(12.08,12.29)	11.99(11.77,12.19)
Hani	-6.3	14.42(14.25,14.58)	13.40(13.24,13.57)	-	13.37(12.88,13.84)	13.12(12.46,13.78)	13.06(12.28,13.83)	12.63(12.48,12.79)
Tu	-6	14.22(14.02,14.43)	14.03(13.80,14.26)	13.63(13.46,13.81)	13.61(12.89,14.33)	13.40(12.76,13.99)	13.09(12.54,13.62)	12.52(12.34,12.70)
Qiang	-6	14.07(13.91,14.23)	13.81(13.63,13.99)	12.49(11.67,13.11)	13.42(13.28,13.56)	12.85(12.71,13.00)	13.15(13.01,13.28)	12.36(12.09,12.64)
Yao	-5.7	14.43(14.22,14.64)	13.59(13.38,13.79)	13.54(13.24,13.95)	12.67(11.75,13.52)	13.41(12.81,13.99)	13.05(12.65,13.44)	12.82(11.64,13.84)
Tujia	-5.4	14.05(13.90,14.20)	-	13.63(13.47,13.78)	11.90(11.36,12.40)	13.02(12.27,13.72)	12.38(12.24,12.52)	12.51(11.54,13.41)
Wa	-5.4	13.85(13.63,14.08)	12.56(12.34,12.78)	-	13.17(11.77,14.58)	13.06(12.91,13.20)	12.80(12.67,12.94)	12.32(12.04,12.60)
Sala	-5.4	14.47(14.18,14.76)	14.26(13.87,14.67)	14.07(13.82,14.31)	14.30(14.15,14.44)	14.32(14.02,14.61)	13.86(13.56,14.14)	12.95(12.77,13.13)
Bai	-5.3	13.91(13.73,14.09)	-	13.44(13.07,13.84)	12.89(11.85,13.89)	12.79(11.77,13.74)	12.40(12.27,12.54)	12.40(11.98,12.78)
Uighur	-4.8	14.21(14.04,14.38)	13.57(13.36,13.78)	13.47(13.26,13.72)	-	13.56(12.92,14.12)	13.37(13.26,13.47)	12.84(12.14,13.44)
Buyi	-4.7	13.91(13.68,14.13)	13.37(13.08,13.66)	13.25(12.93,13.69)	13.12(10.70,15.60)	13.26(13.01,13.51)	12.79(12.48,13.09)	12.59(12.45,12.74)
Miao	-4.6	13.71(13.53,13.89)	13.54(13.33,13.75)	12.95(12.43,13.39)	13.00(12.83,13.18)	13.36(12.99,13.72)	12.63(12.31,12.95)	12.41(12.10,12.71)
Shui	-4.4	-	-	-	13.37(13.07,13.67)	14.02(13.74,14.28)	13.56(13.39,13.72)	12.86(12.22,13.48)
Kirghiz	-4.4	14.72(14.49,14.95)	14.65(14.44,14.86)	13.86(13.67,14.03)	-	13.40(12.81,13.96)	-	13.48(13.34,13.61)
Hui	-4.3	13.73(13.55,13.91)	13.46(13.30,13.62)	13.44(12.63,14.18)	13.28(13.00,13.54)	13.09(12.42,13.76)	12.59(11.76,13.26)	12.51(12.22,12.80)
Korean	-4.2	13.36(13.25,13.47)	12.75(12.58,12.92)	12.59(12.21,12.96)	12.35(12.21,12.49)	11.79(11.68,11.89)	11.96(11.86,12.06)	12.16(10.84,13.16)
Zhuang	-4.1	13.21(13.05,13.36)	13.83(13.66,14.00)	12.86(12.52,13.18)	12.71(12.38,13.04)	12.52(12.33,12.70)	12.12(11.44,12.75)	12.04(11.94,12.13)
Li	-3.6	13.10(12.94,13.26)	13.04(12.84,13.24)	13.23(12.87,13.56)	-	12.95(12.80,13.09)	11.74(11.25,12.20)	12.09(11.94,12.23)
Naxi	-3.3	13.35(13.20,13.51)	13.04(12.86,13.22)	-	11.97(11.83,12.11)	12.64(12.48,12.79)	12.50(12.30,12.70)	12.42(11.92,12.90)
Tibetan	-2.6	13.63(13.42,13.84)	13.72(13.52,13.92)	13.04(12.72,13.39)	13.42(12.84,14.00)	13.12(11.41,14.81)	12.93(12.56,13.29)	12.88(11.81,13.84)
Yi	-1.3	13.80(13.60,14.01)	-	-	-	13.74(13.47,14.00)	13.45(13.30,13.61)	13.42(13.25,13.58)

Table S3 Classification of developmental trajectories of AAM disparities between 25 ethnic minority groups and Han peers based on multi-dimensional clustering (1985–2019)

Ethnic Group	Han Province	Initial Gap [a]	Final Gap [b]	Total Change (Years)[c]	Recent Slope [d]
		I. Rapid Catch-up (Converging, N=13)			
Lisu	Yunnan	1.45 (1985)	0.27	-1.18	-0.02
Dai	Yunnan	0.89 (1985)	0.14	-0.75	0.003
Hani	Yunnan	1.04 (1985)	0.52	-0.52	0
Mongolian	Inner Mongolia	0.48 (1985)	0.03	-0.45	0.019
Qiang	Sichuan	0.60 (1995)	0.24	-0.36	0.021
Kazakh	Xinjiang	0.76 (1995)	0.41	-0.35	0.029
Li	Hainan	0.47 (1995)	0.13	-0.34	-0.034
Wa	Yunnan	0.47 (1985)	0.21	-0.26	-0.027
Bai	Yunnan	0.53 (1985)	0.29	-0.24	0.014
Tu	Qinghai	0.23 (1985)	0	-0.23	-0.052
Dong	Guizhou	0.43 (1985)	0.2	-0.23	0.015
Tujia	Hunan	0.49 (1985)	0.28	-0.21	-0.022
Hui	Ningxia	0.25 (1985)	0.07	-0.18	-0.005
		II. Emerging Divergence (N=8)			
Miao	Guizhou	-0.12 (1985)	0.48	0.6	-0.004
Buyi	Guizhou	0.08 (1985)	0.66	0.58	0.026
Yi	Sichuan	0.89 (2010)	1.3	0.41	0.045
Zhuang	Guangxi	-0.28 (1985)	0.11	0.39	-0.021
Shui	Guizhou	0.55 (2005)	0.93	0.38	-0.03
Korean	Jilin	-0.15 (1985)	0.21	0.36	0.124
Naxi	Yunnan	-0.03 (1985)	0.31	0.34	0.032
Dongxiang	Gansu	1.29 (2010)	1.22	-0.07	-0.005
		III. Fluctuating / Stable (N=4)			
Uighur	Xinjiang	0.51 (1995)	0.69	0.18	-0.022
Sala	Qinghai	0.48 (1985)	0.43	-0.05	-0.107
Yao	Guangxi	0.94 (1985)	0.89	-0.05	-0.034
Kirghiz	Xinjiang	1.59 (1995)	1.33	-0.26	0.067

Note: Groups were clustered using K-Means based on gap trajectories.

[a] Initial Gap: Difference in median AAM (Minority - Han) at the earliest available survey year.

[b] Final Gap: Difference in median AAM in 2019.

[c] Total Change: Final Gap minus Initial Gap; negative values indicate convergence.

[d] Recent Slope: The regression slope of the disparity trend from 2010 to 2019.

Table S4 Parameters of nonlinear (quadratic) regression models associating median AAM with log-transformed per capita GDP among ethnic minorities in China (1995–2019)

	N	R^2	Model *P*	Quadratic Coef	Quadratic *P*
Year-specific Model					
1995	10	0.253	0.36	-0.858	0.325
2000	17	0.118	0.416	-0.242	0.624
2005	20	0.003	0.974	-0.099	0.868
2010	26	0.382	0.004	0.341	0.21
2014	25	0.241	0.048	-0.047	0.888
2019	26	0.307	0.015	0.329	0.325
Pooled Models (1995–2019)					
Unadjusted Model	124	0.353	<0.001	-0.109	0.006
Adjusted Model	124	0.566	<0.001	-0.092	0.032

Note: For the pooled adjusted model, the reported value is the adjusted R^2; all other values are R^2.

Table S5 Mean per capita GDP of residential prefecture for 26 ethnic minority groups in selected survey years (1995–2019)

Ethnic Group	Han Province	1995	2000	2005	2010	2014	2019
I. Rapid Catch-up (Converging)							
Li	Hainan	4,612	8,230	15,034	42,365	54,584	87,105
Mongolian	Inner Mongolia	3,570	7,259	17,773	46,918	85,754	77,039
Hui	Ningxia	4,803	6,143	11,137	25,225	40,750	54,223
Kazakh	Xinjiang	3,825	6,245	10,879	22,005	35,932	51,524
Dai	Yunnan	3,582	5,422	7,459	14,503	26,507	47,659
Hani	Yunnan	2,239	3,663	7,227	14,546	24,473	46,475
Qiang	Sichuan	3,236	4,288	8,488	14,116	27,043	41,278
Bai	Yunnan	2,331	4,030	7,400	15,420	25,593	40,435
Tu	Qinghai	–	3,138	5,885	13,240	26,553	34,705
Lisu	Yunnan	1,306	2,402	4,984	10,331	18,540	34,686
Dong	Guizhou	–	1,805	3,304	8,839	17,807	31,678
Wa	Yunnan	1,534	2,471	4,103	8,988	18,710	29,926
Tujia	Hunan	1,962	2,676	5,026	11,991	18,349	26,691
II. Emerging Divergence							
Shui	Guizhou	–	2,685	4,256	10,861	23,229	46,048
Buyi	Guizhou	–	2,036	3,864	10,839	21,929	44,212
Naxi	Yunnan	–	2,811	5,327	11,680	20,663	36,369
Korean	Jilin	4,355	5,829	9,749	24,448	39,410	34,789
Zhuang	Guangxi	–	7,465	6,415	16,106	25,806	34,194
Yi	Sichuan	2,267	3,630	6,934	17,253	28,556	34,085
Miao	Guizhou	–	1,805	3,304	8,839	17,807	31,678
Dongxiang	Gansu	1,027	1,435	2,893	5,441	10,166	14,697
III. Fluctuating / Stable							
Sala	Qinghai	–	3,138	5,885	13,240	26,553	34,705
Kirghiz	Xinjiang	–	1,832	3,654	7,202	15,222	25,556
Yao	Guangxi	2,697	3,744	5,405	12,991	17,467	24,703
Uighur	Xinjiang	–	2,241	3,941	8,748	16,024	22,647

Checklist S1 GRABDROP reporting table

GRABDROP item 1

Please list all papers published by each co-author in the previous three years that were based on secondary analysis of a big data repository.

**Co-author**	**Eligible publications**
Di Shi	P2, P4, P5, P8, P11, P20, P21, P22, P26, P30, P32, P37, P39, P40, P41, P42
Yunfei Liu	P2, P4, P5, P8, P11, P12, P14, P16, P20, P21, P22, P24, P26, P27, P29, P34, P35, P37, P38, P41, P42, P43, P44, P45, P46
Jiajia Dang	P2, P4, P5, P8, P11, P12, P16, P20, P21, P22, P24, P26, P27, P29, P32, P34, P35, P37, P38, P41, P42, P43, P44, P45, P46
Shan Cai	P2, P4, P5, P8, P11, P16, P20, P21, P22, P24, P26, P29, P31, P34, P35, P37, P38, P41, P43, P44, P45, P46
Yaqi Wang	P1, P6, P9, P10, P11, P13, P15, P19, P21, P23, P30, P32, P36, P39, P40, P41, P42
Jianhui Guo	P1, P6, P9, P10, P13, P14, P18, P19, P21, P23, P30, P32, P36, P39, P40, P41, P42
Xinyao Lian	P1, P6, P9, P10, P13, P19, P21, P23, P30, P32, P36, P39, P40, P41, P42
Shuyue Li	P39, P40
Junyu Lu	P19, P39, P40
Tianyu Huang	P8, P16, P21, P22, P24, P29, P34, P35, P38, P41, P43, P44, P45, P46
Jiaxin Li	P8, P16, P21, P22, P24, P29, P34, P35, P38, P41, P43, P44, P45, P46
Ruolan Yang	P24, P34, P35, P38, P43, P44
Qiuyuan Chen	P35, P38
Peijin Hu	P2, P8, P11, P12, P24, P27, P28, P34, P35, P37, P38, P41, P46
Jun Ma	P2, P3, P4, P5, P7, P8, P11, P12, P16, P17, P20, P21, P22, P24, P25, P26, P27, P28, P33, P34, P35, P37, P38, P41, P43, P46
Jing Li	P1, P6, P9, P10, P11, P13, P14, P15, P18, P19, P21, P23, P29, P30, P32, P36, P37, P39, P40, P41, P42, P45
Yi Song	P2, P3, P4, P5, P7, P8, P11, P12, P14, P16, P17, P20, P21, P22, P24, P25, P26, P27, P28, P29, P30, P31, P32, P33, P34, P35, P37, P38, P41, P42, P43, P44, P45, P46

GRABDROP item	Authors' response
2. Please explain the key elements of your study design and the use of the available datasets that make your study an original scientific contribution.	This study analysed seven repeated cross-sectional waves of the CNSSCH spanning 1985–2019 and included girls from 26 ethnic minority groups. It extends previous work by incorporating the 2019 survey, comparing 25 minority groups with local Han populations, characterising heterogeneous long-term minority–Han difference trajectories, and examining associations with regional per capita GDP.
3. Please list all publications that addressed similar research questions in the same dataset and indicate where you cited them in your paper.	Related CNSSCH studies cited in the manuscript include Lei et al., who examined changes in age at menarche among Han girls and girls from 21 ethnic minority groups using data from 2005 to 2014; Ma et al., who examined long-term trends in age at menarche among Han Chinese girls from 1985 to 2019; and Shi et al., who examined the association between body mass index and pubertal timing using successive CNSSCH waves. These studies are cited in the Introduction and Methods. The present study extends this work by including girls from 26 ethnic minority groups through 2019, comparing minority groups with local Han populations, characterising heterogeneous long-term minority–Han difference trajectories, and examining their associations with regional per capita GDP.
4. Please explain how you addressed multiple testing through an appropriately rigorous statistical threshold and indicate this in the Methods section.	The principal multiple-testing family comprised the 25 prespecified comparisons between individual ethnic minority groups and their corresponding local Han populations in 2019. Two-sided Z-test P values were adjusted using the Benjamini–Hochberg false discovery rate procedure. A q value of <0.05 was considered statistically significant. Twenty-three comparisons remained significant after correction; the Mongolian–Han and Tu–Han comparisons were not significant.
5. Please declare to what extent AI chatbots were used in developing your paper and to which parts of the paper they contributed.	Generative artificial intelligence tools were used solely to assist with language editing and manuscript formatting. They were not used to design the study, generate or analyse data, conduct the statistical analyses, or determine the scientific conclusions. All AI-assisted revisions were critically reviewed and verified by the authors, who take full responsibility for the final manuscript.

Numbered publications

P1. Guo J, Zhou J, Han R, Wang Y, Lian X, Tang Z, et al. Association of Short-Term Co-Exposure to Particulate Matter and Ozone with Mortality Risk. Environ Sci Technol. 2023;57(42):15825-15834. doi:10.1021/acs.est.3c04056.

P2. Cai S, Liu Y, Dang J, Zhong P, Shi D, Chen Z, et al. Clustering of Multilevel Factors Among Children and Adolescents: Associations With Health-Related Physical Fitness. J Phys Act Health. 2024;21(1):29-39. doi:10.1123/jpah.2023-0051.

P3. Chen L, Qin Y, Zhang Y, Song X, Wang R, Jiang J, et al. Association of the external environmental exposome and obesity: A comprehensive nationwide study in 2019 among Chinese children and adolescents. Sci Total Environ. 2024;927:172233. doi:10.1016/j.scitotenv.2024.172233.

P4. Dang J, Cai S, Zhong P, Liu Y, Shi D, Chen Z, et al. Associations of school physical activity environment with comorbid obesity and myopia in children and adolescents: Findings from a Chinese national follow-up study. Scand J Med Sci Sports. 2024;34(1):e14562. doi:10.1111/sms.14562.

P5. Di S, Ning M, Yunfei L, Jiajia D, Panliang Z, Shan C, et al. Association between BMI and age at menarche or spermarche among both sexes: Findings from six successive national surveys in China. J Glob Health. 2024;14:04099. doi:10.7189/jogh.14.04099.

P6. Guo J, Garshick E, Si F, Tang Z, Lian X, Wang Y, et al. Environmental Toxicant Exposure and Depressive Symptoms. JAMA Netw Open. 2024;7(7):e2420259. doi:10.1001/jamanetworkopen.2024.20259.

P7. Guo T, Chen T, Chen L, Liu J, Song X, Zhang Y, et al. Association Between Long Term Exposure to PM(2.5) and Its Components on Severe Obesity in Chinese Children and Adolescents: A National Study in China. Children (Basel). 2024;11(12). doi:10.3390/children11121536.

P8. Li J, Cai S, Liu Y, Dang J, Shi D, Chen Z, et al. Mapping Adolescent Mental Well-Being — 30 PLADs, China, 2019. China CDC Wkly. 2024;6(29):703-707. doi:10.46234/ccdcw2024.159.

P9. Lian X, Guo J, Wang Y, Wang S, Li J. Association between Volatile Organic Compound Exposure and Sex Hormones in Adolescents: The Mediating Role of Serum Albumin. Toxics. 2024;12(6). doi:10.3390/toxics12060438.

P10. Lian X, Wang Y, Guo J, Wan X, Ye X, Zhou J, et al. The short-term effects of individual and mixed ambient air pollutants on suicide mortality: A case-crossover study. J Hazard Mater. 2024;472:134505. doi:10.1016/j.jhazmat.2024.134505.

P11. Liu Y, Wang Y, Xing Y, Wolters M, Shi D, Zhang P, et al. Establish a noninvasive model to screen metabolic dysfunction-associated steatotic liver disease in children aged 6-14 years in China and its applications in high-obesity-risk countries and regions. Lancet Reg Health West Pac. 2024;49:101150. doi:10.1016/j.lanwpc.2024.101150.

P12. Song X, Zhou B, Baird S, Lu C, Ezzati M, Chen L, et al. Trends and inequalities in thinness and obesity among Chinese children and adolescents: evidence from seven national school surveys between 1985 and 2019. Lancet Public Health. 2024;9(12):e1025-e1036. doi:10.1016/S2468-2667(24)00211-1.

P13. Tang Z, Guo J, Zhou J, Yu H, Wang Y, Lian X, et al. The impact of short-term exposures to ambient NO2, O3, and their combined oxidative potential on daily mortality. Environ Res. 2024;241:117634. doi:10.1016/j.envres.2023.117634.

P14. Wang H, Yu X, Guo J, Ma S, Liu Y, Hu Y, et al. Burden of cardiovascular disease among the Western Pacific region and its association with human resources for health, 1990-2021: a systematic analysis of the Global Burden of Disease Study 2021. Lancet Reg Health West Pac. 2024;51:101195. doi:10.1016/j.lanwpc.2024.101195.

P15. Wang Y, Li W, Chen S, Zhang J, Liu X, Jiang J, et al. PM2.5 constituents associated with childhood obesity and larger BMI growth trajectory: A 14-year longitudinal study. Environ Int. 2024;183:108417. doi:10.1016/j.envint.2024.108417.

P16. Cai S, Zhang Y, Chen Z, Liu Y, Dang J, Li J, et al. Secular trends in physical fitness and cardiovascular risks among Chinese college students: an analysis of five successive national surveys between 2000 and 2019. Lancet Reg Health West Pac. 2025;58:101560. doi:10.1016/j.lanwpc.2025.101560.

P17. Chen L, Liu D, Guo Y, Wen B, Wu Y, Xing Y, et al. Impact of climate change and extreme temperature on the incidence of infectious disease among children and adolescents in China: A nationwide case-crossover study with over 8.7 million cases between 2008 and 2019. J Infect. 2025;91(2):106547. doi:10.1016/j.jinf.2025.106547.

P18. Chen M, Wang X, Tan DS, Wang H, Guo J, Li J, et al. Tobacco and alcohol use; suicide ideation, plan, and attempt among adolescents; and the role of legal purchase age restrictions: a pooled population-based analysis from 58 countries. BMC Med. 2025;23(1):163. doi:10.1186/s12916-025-03983-6.

P19. Chen X, Si F, Lian X, et al. Moderating Effects of Physical Activity on PM2.5-Associated Depressive Symptom Risk Among College Students. J Hazard Mater. 2025;500:140354. doi:10.1016/j.jhazmat.2025.140354.

P20. Dang J, Liu Y, Cai S, Zhong P, Shi D, Chen Z, et al. Secular trend and projection of overweight and obesity among Chinese children and adolescents aged 7-18 years from 1985 to 2019: Rural areas are becoming the focus of investment. Chin Med J (Engl). 2025;138(3):311-317. doi:10.1097/CM9.0000000000003123.

P21. Dang J, Wang Y, Ma N, Cai S, Guo J, Liu Y, et al. The impact of long-term exposure to NO(2), O(3), and their oxidative potential on adolescents' mental health, and the protective role of school-based greenness. Environ Int. 2025;195:109212. doi:10.1016/j.envint.2024.109212.

P22. Dang J, Zhang Y, Liu Y, Shi D, Cai S, Chen Z, et al. Spatial-temporal analysis and spatial drivers of childhood obesity in China from 1985 to 2019. Obesity (Silver Spring). 2025;33(7):1344-1354. doi:10.1002/oby.24303.

P23. Guo J, Koutrakis P, Zilli Vieira CL, Fan Y, Wang Y, Lian X, et al. Modifiable influencing factors and their joint effects on early- and late-onset coronary heart disease. Nat Commun. 2025;16(1):10930. doi:10.1038/s41467-025-65963-x.

P24. Huang T, Dang J, Li J, Cai S, Liu Y, Chen Z, et al. Trends and Inequalities of Co-Occurring Obesity and Elevated Blood Pressure Among Chinese Children and Adolescents Aged 7-18 Years from 1985 to 2019 and Projections to 2030. Nutrients. 2025;17(17). doi:10.3390/nu17172828.

P25. Liu J, Song X, Chen M, Zhang Y, Jiang J, Wang R, et al. Modifying effects of compensatory sleep on elevated blood pressure related to school-day sleep patterns. BMC Med. 2025;23(1):663. doi:10.1186/s12916-025-04439-7.

P26. Liu Y, Luo D, Zhong P, Dang J, Shi D, Cai S, et al. Burden and risk factors of premature drowning mortality in 204 countries and territories, 1980-2021. Sci Rep. 2025;15(1):21036. doi:10.1038/s41598-025-05418-x.

P27. Song X, Zhou B, Baird S, Lu C, Song Z, Zhang Y, et al. Trends and predictions to 2030 in demographic structures and metabolic health for children and adolescents in China: analysis of national school health surveys from 2000 to 2019. Lancet Child Adolesc Health. 2025;9(8):530-543. doi:10.1016/S2352-4642(25)00140-3.

P28. Song Z, Song X, Chen L, Jiang J, Zhang Y, Liu J, et al. Environmental greenness, physical activity, and their synergistic effects on vital capacity weight index in children and adolescents exposed to PM(2.5) and O(3) in economically developed provinces of China. BMC Public Health. 2025;25(1):3427. doi:10.1186/s12889-025-24439-9.
P29. Sun Z, Yang Y, Zhong X, Dang J, Cai S, Liu Y, et al. How Family and Individual Physical Activity Environments Relate to Obesity Transition in Chinese Children and Adolescents. Nutrients. 2025;17(23). doi:10.3390/nu17233760.
P30. Wang F, Lian X, Wang Y, Wang H, Guo J, Tang Z, et al. Short-term exposure to PM2.5 and high pollution events on depressive symptoms among adolescents. J Hazard Mater. 2025;492:138131. doi:10.1016/j.jhazmat.2025.138131.
P31. Wang X, Wang H, Yuan X, Cai S, Huang Y, Song Y, et al. Imbalance between muscle strength development and weight gain in children and young adults in China: serial cross-sectional evidence from 1.33 million students from five successive national surveys between 2000 and 2019. Lancet Reg Health West Pac. 2025;61:101640. doi:10.1016/j.lanwpc.2025.101640.
P32. Wang Y, Shi D, Ye X, Dang J, Guo J, Lian X, et al. Spatiotemporal Patterns of Indoor Air Pollution and Its Association with Depressive Symptoms Among Schoolchildren in China. Toxics. 2025;13(7):563. doi:10.3390/toxics13070563.
P33. Yu X, Wang H, Ma S, Dong Y, Ma Y, Song Y, et al. Impact of parental myopia on myopia in schoolchildren and adolescents in China: A national cross-sectional survey. Chin Med J (Engl). 2025. doi:10.1097/CM9.0000000000003515.
P34. Cai S, Li J, Huang T, Liu Y, Dang J, Chen Z, et al. Changing landscape of physical fitness and urban-rural inequalities: evidence from 1.2 million children and adolescents over 20 years of urbanization. BMC Med. 2026;24(1). doi:10.1186/s12916-026-04712-3.
P35. Cai S, Li L, Huang T, Liu Y, Dang J, Li J, et al. Mapping transitions and inequalities in the burden of malnutrition among Chinese children and adolescents: a dual-level analysis of 6 nationwide surveys, 1995 to 2019. Am J Clin Nutr. 2026;124(1):101354. doi:10.1016/j.ajcnut.2026.101354.
P36. Guo J, Zhong X, Koutrakis P, Vieira CLZ, Si F, Wang Y, et al. Long-Term Ambient Benzene Exposure and Brain Disorders Among Urban Adults: Effect Modification by Genetic Susceptibility and Potential Mediation by Plasma Proteins. Adv Sci (Weinh). 2026:e75874. doi:10.1002/advs.75874.
P37. Hao M, Shi D, Liu Y, Dang J, Cai S, Shen Y, et al. Spatiotemporal Patterns of Age at Menarche or Spermarche in Chinese Children and Adolescents and Socioeconomic and Environmental Determinants: Findings from a Series of Nation-Wide Surveys, 1995 to 2019. Environ Health (Wash). 2026. doi:10.1021/envhealth.5c00793.
P38. Huang T, Dang J, Cai S, Li J, Liu Y, Yang R, et al. Transitions From Overweight to Obesity and the Rising Severe Obesity Burden Among Children and Adolescents in China. Obesity (Silver Spring). 2026;34(5):1128-1138. doi:10.1002/oby.70187.
P39. Li S, Guo J, Wang Y, Shi D, Lian X, Lu J, Li J. Inflammatory and metabolic pathways underlying the association between PM2.5 exposure and dementia. Environ Pollut. 2026;397:127961. doi:10.1016/j.envpol.2026.127961.
P40. Lu J, Guo J, Li Y, Shi D, Wang Y, Lian X, et al. Environmental Toxicant Exposure and Height Among Children and Adolescents. Toxics. 2026;14(6).

doi:10.3390/toxics14060481.
P41. Wang Y, Dang J, Guo J, Shi D, Ma N, Lian X, et al. Extreme Cold Events and Adolescent Mental Health: Evidence from a National School-Based Survey in China. Environ Health (Wash). 2026;4(3):502-512. doi:10.1021/envhealth.5c00287.
P42. Wang Y, Shi D, Dang J, Liu J, Liu Y, Chen Z, et al. Impact of Outdoor Environmental Exposome on Depressive Symptoms Among Children Aged 9-14 in China. Environ Sci Technol. 2026;60(6):4565-4578. doi:10.1021/acs.est.5c10618.
P43. Yang R, Cai S, Dang J, Huang T, Li J, Liu Y, et al. Associations of the Muscle Strength Index with Overweight/Obesity, Elevated Blood Pressure, and Their Comorbidity in Chinese Children and Adolescents During Two Decades. J Clin Med. 2026;15(7). doi:10.3390/jcm15072712.
P44. Yang Y, Sun Z, Cai S, Dang J, Liu Y, Li J, et al. The High Prevalence of High Normal Blood Pressure and the Differential Association of Grip Strength Metrics with Hypertension in a National Sample of Chinese College Students. J Clin Med. 2026;15(3):992. doi:10.3390/jcm15030992.
P45. Yang Y, Sun Z, Zhong X, Dang J, Cai S, Liu Y, et al. Association Between Outdoor Physical Activity and Height Growth Velocity in Chinese Children Aged 9-15: A Secondary Analysis of a National Population-Based Cohort. Healthcare (Basel). 2026;14(5). doi:10.3390/healthcare14050628.
P46. Zhang Y, Cai S, Huang T, Li J, Dang J, Chen Z, et al. Evaluating WHtR and WC cut-offs for abdominal obesity among children and adolescents: Insights from three National Surveys in China. Diabetes Obes Metab. 2026;28(4):3011-3019. doi:10.1111/dom.70488.

Checklist S2 STROBE Statement—Checklist of items that should be included in reports of cross-sectional studies

	Item No	Recommendation	Location in current manuscript
Title and abstract	1	(a) Indicate the study's design with a commonly used term in the title or the abstract	Title and structured abstract
		(b) Provide in the abstract an informative and balanced summary of what was done and what was found	Structured abstract
Introduction			
Background/rationale	2	Explain the scientific background and rationale for the investigation being reported	Introduction, paragraphs 1–2
Objectives	3	State specific objectives, including any prespecified hypotheses	Introduction, final paragraph
Methods			
Study design	4	Present key elements of study design early in the paper	Methods—Subjects, paragraph 1
Setting	5	Describe the setting, locations, and relevant dates, including periods of recruitment, exposure, follow-up, and data collection	Methods—Subjects, paragraphs 1–3
Participants	6	(a) Give the eligibility criteria, and the sources and methods of selection of participants	Methods—Subjects and Measures
Variables	7	Clearly define all outcomes, exposures, predictors, potential confounders, and effect modifiers. Give diagnostic criteria, if applicable	Methods—Measures
Data sources/ measurement	8*	For each variable of interest, give sources of data and details of methods of assessment (measurement). Describe comparability of assessment methods if there is more than one group	Methods—Measures and Statistical analysis
Bias	9	Describe any efforts to address potential sources of bias	Methods—standardised survey procedures; Discussion—limitations

Study size	10	Explain how the study size was arrived at	Methods—Measures: all eligible participants with available data; analytic sample n=190,064
Quantitative variables	11	Explain how quantitative variables were handled in the analyses. If applicable, describe which groupings were chosen and why	Methods—Statistical analysis: age intervals, log-transformed GDP, trajectory features
Statistical methods	12	(a) Describe all statistical methods, including those used to control for confounding	Methods—Statistical analysis
		(b) Describe any methods used to examine subgroups and interactions	Methods—ethnic group-specific analyses and pooled GDP model; no formal interaction analysis
		(c) Explain how missing data were addressed	Methods—Measures: complete-case exclusion for unavailable GDP/AAM data; no imputation
		(d) If applicable, describe analytical methods taking account of sampling strategy	Methods—Statistical analysis: sampling weights and school-level clustering were not incorporated into the analyses
		(e) Describe any sensitivity analyses	N/A—no formal sensitivity analyses were conducted
Results			
Participants	13*	(a) Report numbers of individuals at each stage of study—eg numbers potentially eligible, examined for eligibility, confirmed eligible, included in the study, completing follow-up, and analysed	Methods—Measures; Table S1; Figure S1
		(b) Give reasons for non-participation at each stage	Methods—Subjects and Measures; Figure S1
		(c) Consider use of a flow diagram	Figure S1 in the Online Supplementary Document
Descriptive data	14*	(a) Give characteristics of study participants (eg demographic, clinical, social) and information on exposures and potential confounders	Table S1; Results
		(b) Indicate number of participants with missing data for each variable of interest	Methods—Subjects/Measures; unavailable ethnic group–survey wave combinations reported;

Outcome data	15*	Report numbers of outcome events or summary measures	Results—Menarche status in ethnic minorities in 2019; Figure 1
Main results	16	(a) Give unadjusted estimates and, if applicable, confounder-adjusted estimates and their precision (eg, 95% confidence interval). Make clear which confounders were adjusted for and why they were included	Results; Figures 1-3; Tables S1–S5
		(b) Report category boundaries when continuous variables were categorized	Methods—trajectory definitions; Table S3
		(c) If relevant, consider translating estimates of relative risk into absolute risk for a meaningful time period	N/A
Other analyses	17	Report other analyses done—eg analyses of subgroups and interactions, and sensitivity analyses	Results—trajectory clustering and GDP analyses
Discussion			
Key results	18	Summarise key results with reference to study objectives	Discussion, paragraph 1
Limitations	19	Discuss limitations of the study, taking into account sources of potential bias or imprecision. Discuss both direction and magnitude of any potential bias	Discussion—strengths and limitations
Interpretation	20	Give a cautious overall interpretation of results considering objectives, limitations, multiplicity of analyses, results from similar studies, and other relevant evidence	Discussion
Generalisability	21	Discuss the generalisability (external validity) of the study results	Discussion—limitations and interpretation
Other information			
Funding	22	Give the source of funding and the role of the funders for the present study and, if applicable, for the original study on which the present article is based	Funding statement
